# Verbascoside-Rich *Abeliophyllum distichum* Nakai Leaf Extracts Prevent LPS-Induced Preterm Birth Through Inhibiting the Expression of Proinflammatory Cytokines from Macrophages and the Cell Death of Trophoblasts Induced by TNF-α

**DOI:** 10.3390/molecules25194579

**Published:** 2020-10-07

**Authors:** Ho Won Kim, A-Reum Yu, Minji Kang, Nak-Yun Sung, Byung Soo Lee, Sang-Yun Park, In-Jun Han, Dong-Sub Kim, Sang-Muk Oh, Young Ik Lee, Gunho Won, Sung Ki Lee, Jong-Seok Kim

**Affiliations:** 1 Myunggok Medical Research Institute, College of Medicine, Konyang University, Daejeon 35365, Korea; kimong104@naver.com (H.W.K.); kyoaor22@hanmail.net (A.-R.Y.); 2 Department of Medical Science, Chungnam National University, Daejeon 35365, Korea; mjkangs92@naver.com; 3 Division of Natural Product Research, Korea Prime Pharmacy CO. LTD., Jeonnam 58144, Korea; ny.sung@koreaprime.co.kr (N.-Y.S.); bs.lee@koreaprime.co.kr (B.S.L.); sy.park@koreaprime.co.kr (S.-Y.P.); ij.han@koreaprime.co.kr (I.-J.H.); ds.kim@koreaprime.co.kr (D.-S.K.); 4 Department of Biochemistry, College of Medicine, Konyang University, Daejeon 35365, Korea; sangmuk_oh@konyang.ac.kr; 5 Industrial Bioresource Research Center, Korea Research Institute of Bioscience and Biotechnology (KRIBB), Daejeon 35365, Korea; yilee@kribb.re.kr; 6 Centers for Disease Control & Prevention National Institute of Health 187, Chungcheongbuk-do 28159, Korea; wgh007@korea.kr; 7 Department of Obstetrics and Gynecology, Konyang University Hospital, Daejeon 35365, Korea

**Keywords:** *Abeliophyllum distichum* Nakai, preterm birth, inflammation, TNF-α, macrophage, trophoblast

## Abstract

*Background*: Preterm birth is a known leading cause of neonatal mortality and morbidity. The underlying causes of pregnancy-associated complications are numerous, but infection and inflammation are the essential high-risk factors. However, there are no safe and effective preventive drugs that can be applied to pregnant women. *Objective*: The objectives of the study were to investigate a natural product, *Abeliophyllum distichum* leaf (ADL) extract, to examine the possibility of preventing preterm birth caused by inflammation. Methods: We used a mouse preterm birth model by intraperitoneally injecting lipopolysaccharides (LPS). ELISA, Western blot, real-time PCR and immunofluorescence staining analyses were performed to confirm the anti-inflammatory efficacy and related mechanisms of the ADL extracts. Cytotoxicity and cell death were measured using Cell Counting Kit-8 (CCK-8) analysis and flow cytometer. *Results*: A daily administration of ADL extract significantly reduced preterm birth, fetal loss, and fetal growth restriction after an intraperitoneal injection of LPS in mice. The ADL extract prevented the LPS-induced expression of TNF-α in maternal serum and amniotic fluid and attenuated the LPS-induced upregulation of placental proinflammatory genes, including IL-1β, IL-6, IL-12p40, and TNF-α and the chemokine gene CXCL-1, CCL-2, CCL3, and CCL-4. LPS-treated THP-1 cell-conditioned medium accelerated trophoblast cell death, and TNF-α played an essential role in this effect. The ADL extract reduced LPS-treated THP-1 cell-conditioned medium-induced trophoblast cell death by inhibiting MAPKs and the NF-κB pathway in macrophages. ADL extract prevented exogenous TNF-α-induced increased trophoblast cell death and decreased cell viability. *Conclusions*: We have demonstrated that the inhibition of LPS-induced inflammation by ADL extract can prevent preterm birth, fetal loss, and fetal growth restriction.

## 1. Introduction

Inflammation triggered by infection leads to death in newborns, especially premature infants, and poses a substantial health burden worldwide [1]. In humans, Gram-negative bacterial infections are associated with fetal mortality and preterm labor [2]. According to several previous reports, maternal LPS exposure at late gestational stages induces fetal death, intrauterine growth restriction (IUGR), skeletal development retardation, and preterm labor and birth. Maternal LPS exposure induces fetal demise, neural tube defects (NTDs), and IUGR by upregulating proinflammatory cytokines, such as TNF-α and IL-1β [3,4,5,6,7]. Indeed, the production of proinflammatory cytokines, especially TNF-α, and chemokines in the maternal serum and amniotic fluid is elevated following maternal LPS exposure [8].

During pregnancy, macrophages comprise 20–30% of all human decidual leukocytes in the maternal–fetal interface [9]. Macrophages sustain a normal pregnancy by producing various cytokines, promoting trophoblast cell invasion, remodeling spiral arteries, and the phagocytosis of apoptotic cells [10,11,12,13]. Conversely, numerous activated macrophages are observed at the maternal–fetal interface in abnormal pregnancies. These macrophages are associated with an inadequate remodeling of the uterine vessels and defective trophoblast invasion and ultimately lead to spontaneous abortion, preeclampsia, and preterm birth [14,15,16]. Although it has various primary causes, increased inflammation is a shared pathology in preterm birth. Therefore, regulating increases in the inflammatory response is a promising strategy for the treatment of infection-induced preterm birth.

*Abeliophyllum distichum* Nakai (*A. distichum*) is a monotypic genus with a single species of deciduous shrub belonging to the Oleaceae, or olive, family, and it is natively grown in South Korea. Although *A. distichum* is used as a landscape plant, it was recently revealed to exert pharmaceutical properties, such as anticancer, antidiabetic, antihypertensive, and anti-inflammatory activities [17,18]. Therefore, *A. distichum* has potential as a botanical drug and a dietary health supplement. Although the anti-inflammatory properties of *A. distichum* have been investigated, the association of *A. distichum* with inflammation-induced preterm birth remains unknown. In this study, we hypothesized that *A. distichum* may alleviate inflammation-induced preterm birth by inhibiting inflammatory signaling in macrophages. We also investigated the effect of *A. distichum* on the crosstalk between macrophages and trophoblasts, focusing on TNF-α.

## 2. Results

### 2.1. HPLC Analysis of ADL Extract

**Figure 1:**
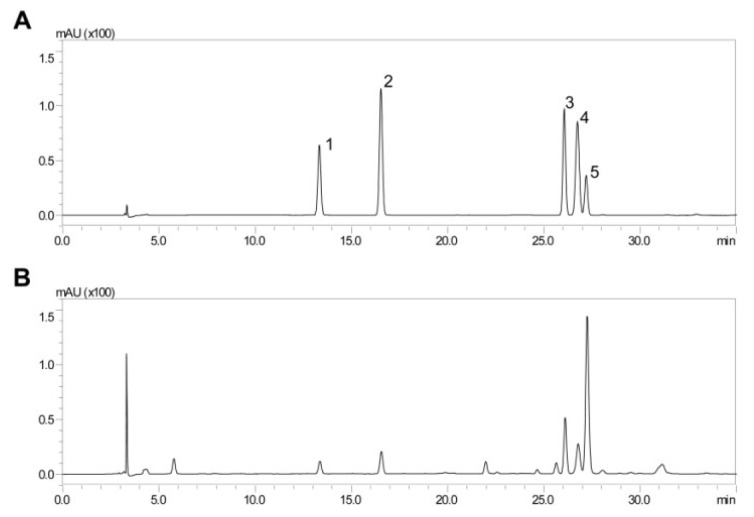
HPLC chromatogram of *A. distichum* ethanolic extract and five standard phenolic compounds. (**A**) The standard phenolic compounds are 1: chlorogenic acid, 2: caffeic acid, 3: rutin, 4: ferulic acid, 5: verbascoside. (**B**) The extracts were analyzed with a Shimadzu liquid chromatography system (LC-20AD) with a column (C18, 4.6 × 250 mm, 5 μm), and the mobile phase consisted of solvents A and B. Solvent A was 0.1% trifluoroacetic acid in water, and solvent B was 0.1% trifluoroacetic acid in acetonitrile. The gradient was 0 min, 10% B; 0–20 min, 20% B; 20–30 min, 20% B; 30–35 min, 100% B; 35–40 min, 100% B; 40–41 min, 10% B; 41–50 min, 10% B. The run time was 50 min, and the flow rate was 1.0 mL/min.

One of the most important benefits of some phenolic compounds is their ability to counter-regulate inflammation [19]. To identify the active compound that exerts anti-inflammatory effects to inhibit LPS-induced abortion, polyphenolic compounds in *Abeliophyllum distichum* leaf (ADL) ethanolic extract were identified and quantified using HPLC analysis (Figure 1A, B). Five polyphenolic compounds, including chlorogenic acid, caffeic acid, rutin, ferulic acid and verbascoside (also named acteoside), were identified in ADL extract (Table 1). As shown in Table 1, 0.26 mg of verbascoside per 1 mg of ADL extracts was contained in a very high proportion as a single substance. 

### 2.2. ADL Extract Prevents LPS-Induced Preterm Birth and Fetal Growth Restriction

**Figure 2:**
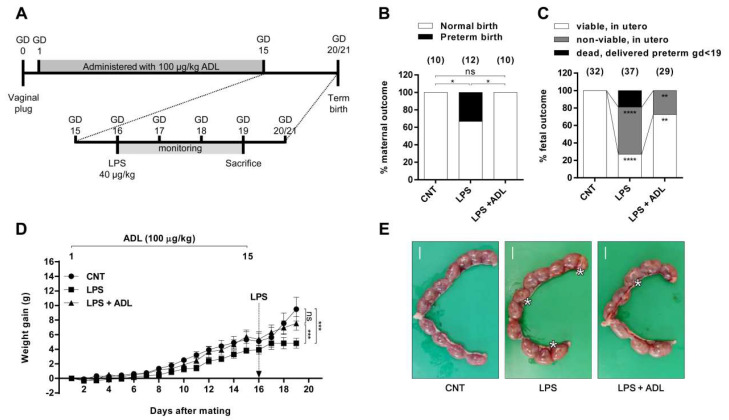
ADL extract prevents LPS-induced preterm birth and fetal loss. (**A**) Schematic illustration of the mouse model of LPS-induced preterm birth and fetal loss. In the LPS+ADL groups, pregnant mice were gavaged with ADL extract (100 µg/kg) daily from GD 1 to GD 15. In the LPS alone and LPS+ADL groups, pregnant mice were i.p. injected with LPS (40 μg/kg) on GD 16 and then observed for preterm birth within 72 h of LPS administration. All pregnant mice were sacrificed on GD 19. (**B**) The ratio of pups associated with different maternal outcomes relative to the total number of pups 72 h after LPS injection. Pregnancy outcomes were classified as normal (>1 live fetus) or preterm (preterm delivery on GD < 19). The number of pregnant mice per group is shown in parentheses. (**C**) The ratio of pups associated with different fetal outcomes relative to the total number of pups 72 h after LPS injection. Fetal outcomes were classified as (i) viable, in utero, (ii) nonviable, in utero, or (iii) dead, delivered preterm (GD < 19). The number of pups per group is shown in parentheses. The Chi^2^ test was used for statistical comparisons (* < 0.05, ** < 0.01, and **** < 0.001). (**D**) Maternal weights were recorded daily from GD 2 to GD 19. The data represent the mean ± standard deviation, n = 4. *** < 0.005 and ns > 0.05 compared with the CNT group. (E) Representative photographs of uterine horns from each group on GD 19 are shown. “*” indicates nonviable fetuses in utero. Scale bar = 10 mm.

**Figure 3:**
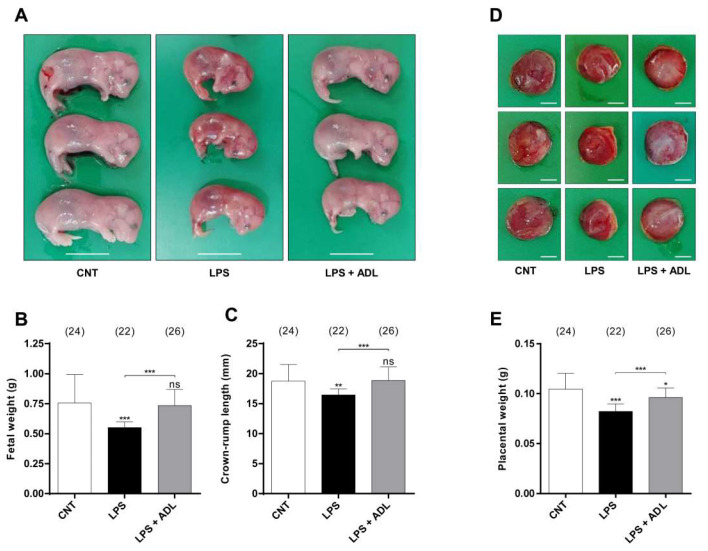
ADL extract pretreatment alleviates LPS-induced fetal growth restriction. In the LPS+ADL groups, pregnant mice were gavaged with ADL extract (100 µg/kg) daily from GD 1 to GD 15. In the LPS alone and LPS+ADL groups, pregnant mice were i.p. injected with LPS (40 μg/kg) on GD 16. All mice were sacrificed on GD 19. Representative photographs of (**A**) fetuses and (**D**) placentas from each group are shown. A: scale bar = 10 mm; D: scale bar = 4 mm. (**B**) The fetal weight, (**C**) crown–rump length, and (**E**) placental weight of mice from the different groups. All data represent the mean ± standard deviation. ns > 0.05, * < 0.05, ** < 0.01, and *** < 0.005 compared with the CNT group.

To investigate whether ADL extract also has a preventive effect against inflammation-induced preterm birth, we administered ADL extract orally for 15 days beginning 1 day after pregnancy. Then, LPS was injected intraperitoneal (i.p.) at a dose of 40 μg/kg on day 16, and the mice were monitored for 3 days (Figure 2A). LPS-treated mice exhibited adverse outcomes, with 33.33% (4/12) of the mice delivering preterm within 72 h of LPS administration (Figure 3B). Interestingly, the pretreatment of pregnant mice with ADL extract reduced the preterm birth rate to 0% (0/10) after LPS administration (Figure 2B). Analysis of the dams on gestational day (GD) 19 revealed adverse outcomes after LPS administration (Figure 2C). The autopsy of the 66.67% (4/6) of LPS-treated dams that remained undelivered on GD 19 revealed a higher incidence of fetal death (*n* = 4, 54.05%, 20/37) in utero than that for control (CNT) mice (*n* = 5, 0%, 0/32) (Figure 2C). Treatment with ADL extract protected LPS-treated mice from remaining undelivered on GD 19, reducing fetal death (*n* = 5, 27.59%, 8/29) in utero (Figure 2C). The injection of LPS prevented pregnant mice from gaining weight. However, compared to LPS alone, ADL extract pretreatment attenuated LPS-induced maternal weight loss (Figure 2D).

In the preterm birth experiment, we analyzed the fetal and placental weight and crown–rump length of fetuses that survived in utero on GD 19. As expected, the fetal and placental weight and crown–rump length were significantly lower in the LPS group (*n* = 4) than the CNT groups (*n* = 5). Interestingly, ADL extract pretreatment (*n* = 5) significantly alleviated the LPS-induced reductions in fetal and placental weight and crown–rump length (Figure 3). These data indicate that ADL extract can effectively inhibit TLR4 signaling to prevent stillbirth elicited by LPS administration in late gestation. Moreover, ADL extract prevents preterm birth and fetal loss and alleviates fetal growth restriction in a physiologically relevant model of preterm birth and in utero inflammation.

### 2.3. ADL Extract Pretreatment Downregulates LPS-Induced Expression of Proinflammatory Cytokines and Chemokines in Placental Tissue

**Figure 4:**
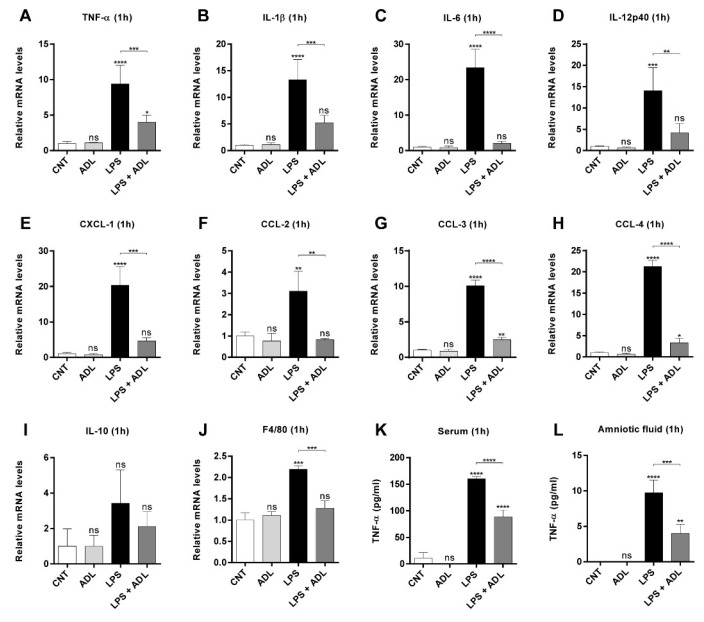
ADL extract prevents LPS-induced expression of proinflammatory cytokines and chemokines. In the ADL alone and LPS+ADL groups, pregnant mice were gavaged with ADL extract (100 μg/kg) daily from GD 1 to GD15. In the LPS alone and LPS+ADL groups, pregnant mice were i.p. injected with LPS (40 μg/kg) on GD 16. Mouse placental tissues were collected 1 h after LPS (40 μg/kg) injection. Placental samples were collected from maternal mice of each group, pooled, and used for analysis. Relative mRNA expression levels of (**A**) TNF-α, (**B**) IL-1β, (**C**) IL-6, (**D**) IL-12p40, (**E**) CXCL-1, (**F**) CCL-2, (**G**) CCL-3, (**H**) CCL-4, (**I**) IL-10 and (**J**) F4/80 in the placenta were measured by real-time RT-PCR and normalized to the level of β-actin. Maternal serum and amniotic fluid were collected 1 h after LPS (40 μg/kg) injection. Amniotic fluid samples were collected from maternal mice from each group, pooled, and used for analysis. The concentration of TNF-α was measured in (K) maternal serum and (L) amniotic fluid using ELISA. All data represent the mean ± standard deviation, n = 3–4. ns > 0.05, * < 0.05, ** < 0.01, *** < 0.005, and **** < 0.001 compared with the CNT group.

Numerous animal experiments have demonstrated that proinflammatory cytokines and chemokines are associated with LPS-induced fetal death, preterm delivery, and IUGR [20,21]. Therefore, we investigated the effect of ADL extract pretreatment on LPS-induced cytokine and chemokine expression in placental tissues. The results showed that an administration of LPS (40 μg/kg) caused a significant increase in the levels of proinflammatory cytokines (TNF-α, IL-1β, IL-6, and IL-12p40) and chemokines (CCL-2, -3, -4, and CXCL-1) in the placenta (Figure 4A–H). However, the administration of ADL extract alone in the absence of LPS did not induce proinflammatory cytokine or chemokine expression (Figure 4A–H). Interestingly, the levels of proinflammatory cytokine and chemokine in the ADL extract-pretreated group decreased compared to those in the LPS-treated group to levels similar to those in the CNT group (Figure 4A–H). In contrast, no statistically significant changes in IL-10 mRNA levels in the placenta were observed 1 h after LPS administration (Figure 4I). In addition, we confirmed that the F4/80 gene in the placenta was increased with LPS treatment and decreased with ADL pretreatment (Figure 4J and Supplementary Figure S1F). These data suggest that LPS treatment increases monocyte–macrophage infiltration in the placenta and that ADL can effectively inhibit the infiltration of these cells.

Only TNF-α mRNA levels remained increased in the placenta at 24 h after LPS administration, while the levels of the other proinflammatory cytokines (IL-1β, IL-6, and IL-12p40) in the same biological specimens almost returned to CNT levels (Supplementary Figure S1A–D). Additionally, in the same samples, no significant changes in CXCL-1 expression were found between the only-LPS-treated and ADL extract-pretreated groups. Therefore, we investigated the effect of ADL extract pretreatment on LPS-induced TNF-α levels in the maternal serum and amniotic fluid. The results showed that compared to control treatment, the administration of LPS (40 μg/kg) significantly elevated the levels of TNF-α in both the serum and amniotic fluid of pregnant mice after 1 h (Figure 4J, K). Interestingly, ADL extract pretreatment reduced the levels of TNF-α in both the serum and amniotic fluid of pregnant mice after LPS administration (Figure 4J, K). Additionally, ADL extract pretreatment similarly reduced the levels of TNF-α in the serum at 24 h after LPS administration (Supplementary Figure S1F). Thus, these results suggested that ADL extract decreases the LPS-induced secretion of proinflammatory cytokines and chemokines, especially TNF-α, in placental tissues, maternal serum, and amniotic fluid.

### 2.4. ADL Extract Inhibits the Production of TNF-α in Macrophages by Regulating Mitogen-Activated Protein Kinases (MAPKs) and NF-κB

**Figure 5:**
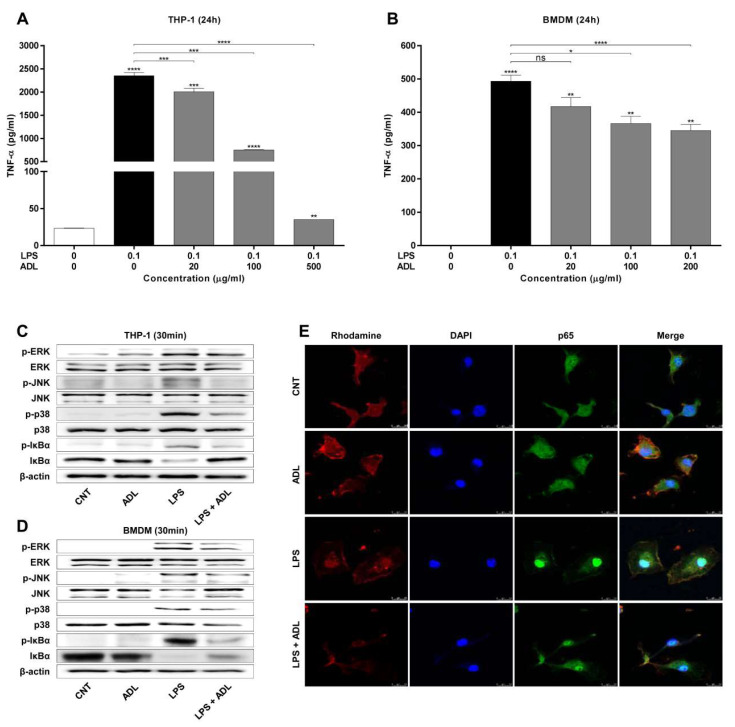
ADL extract inhibits LPS-induced TNF-α production by suppressing MAPK/NF-κB signaling in macrophages. Human promonocytic (THP-1) cells differentiated with phorbol myristic acetate (PMA) (100 μM) overnight were cotreated with LPS (100 ng/mL) and ADL extract (20, 100, or 500 µg/mL) for 24 h. Murine bone marrow-derived macrophages (BMDMs) were cotreated with LPS (100 ng/mL) and ADL extract (20, 100, or 200 μg/mL) for 24 h. (**A**, **B**) The production of TNF-α in the supernatant was measured by ELISA. THP-1 cells and BMDMs were cotreated with LPS (100 ng/mL) and ADL extract (100 μg/mL) for 30 min. The data represent the mean ± standard deviation, n = 3. ns > 0.05, * < 0.05, ** < 0.01, *** < 0.005, and **** < 0.001 compared with the untreated group. (**C**, **D**) Whole-cell lysates were prepared, and phospho-ERK/ERK, phospho-JNK/JNK, phospho-p38/p38, phospho-IκBα/IκBα, and β-actin levels were analyzed by Western blotting. (**E**) Confocal imaging of p65 and 4′,6-diamidino-2-phenylindole (DAPI) were performed. BMDMs were pretreated with ADL extract (100 μg/mL) for 1 h and stimulated with LPS (100 ng/mL) for 30 min. Cells were stained with an Alexa Fluor 488-labeled NF-κB/p65 antibody (green), and the nuclei were stained with DAPI (blue). Images were captured at 100×.

In infection-induced preterm birth, the number of macrophages is increased in the decidua, and macrophages secrete proinflammatory cytokines such as TNF-α and IL-1β [22]. Therefore, we next investigated whether ADL extract affects the production of TNF-α by macrophages. An ELISA revealed that LPS elevated TNF-α levels in both THP-1 cells and bone marrow-derived macrophages (BMDMs) at 24 h. The LPS-induced increase in the levels of TNF-α was significantly inhibited by ADL extract in a dose-dependent manner in both cell types (Figure 5A,B). ADL extract was not observed to induce toxicity in either cell type (Supplementary Figure S2A,B). The inflammatory cytokine TNF-α is modulated by MAPKs and the NF-κB pathway. To determine the mechanism underlying the regulatory effect of ADL extract on MAPKs and the NF-κB pathway, we analyzed the phosphorylation of MAPK and IκBα in THP-1 cells and BMDMs. The phosphorylation levels of ERK and p38 and JNK were increased after 30 min of LPS stimulation. However, cotreatment with ADL extract inhibited the LPS-induced phosphorylation of p38, ERK, and JNK in both cell types. In addition, the LPS-induced phosphorylation of IκBα and subsequent IκBα degradation 30 min after stimulation. As expected, cotreatment with ADL extract blocked LPS-induced IκBα degradation in both cell types (Figure 5C,D). We attempted to confirm the effect of ADL extract on the inhibition of NF-κB nuclear translocation by the immunofluorescence staining of BMDMs following treatment with LPS and ADL extracts. BMDMs were pretreated with ADL extract (100 μg/mL) for 1 h and stimulated with LPS (100 ng/mL) for 30 min. NF-κB p65 was mainly localized in the cytoplasm in the CNT and ADL groups. After 30 min of treatment with 100 ng/mL LPS, widespread nuclear staining of NF-κB p65, which indicates the translocation of NF-κB p65 into the nuclei, was observed. However, pretreatment with 100 μg/mL ADL extract blocked the nuclear translocation of NF-κB p65 from the cytoplasm to the nucleus in both cell types (Figure 5E,F). This result is consistent with the results observed for MAPKs and IκBα, which showed that the activation of NF-κB was suppressed by ADL extract.

### 2.5. ADL Extract Inhibits TNF-α-Induced Trophoblast Cell Death Resulting from Exposure to LPS-Treated THP-1 Cell-Conditioned and Medium

**Figure 6:**
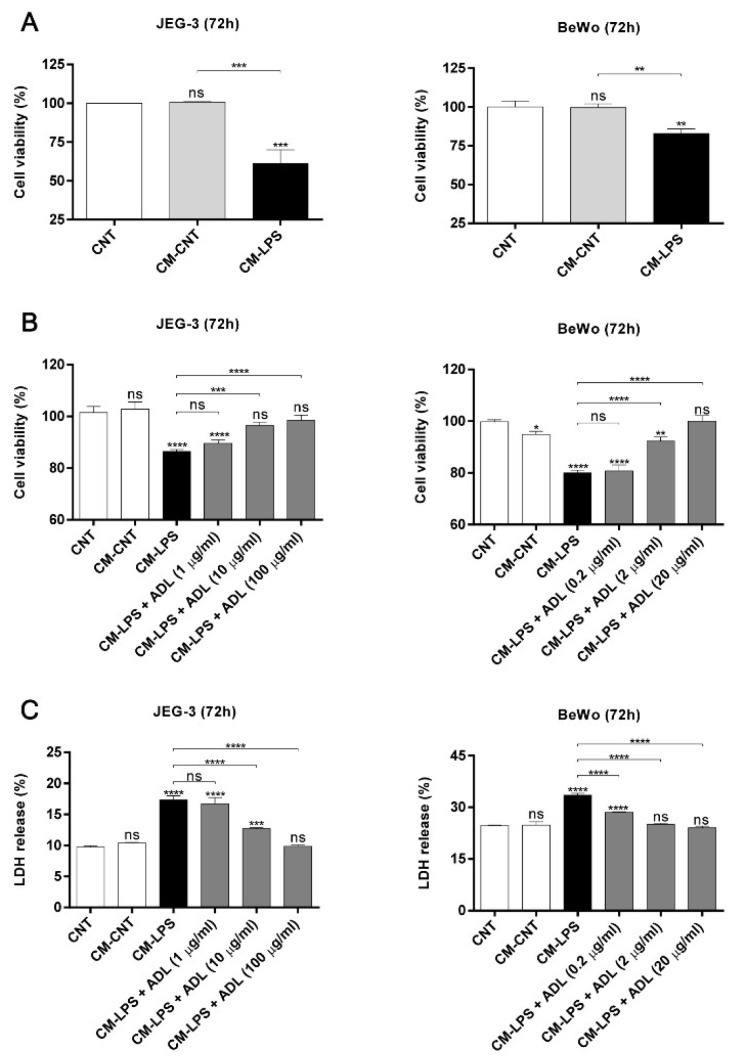
Effects of conditioned medium from LPS-stimulated and ADL extract-treated THP-1 cells on trophoblasts. Trophoblasts were treated with culture medium (CNT), THP-1 cell-conditioned medium (CM-CNT), or LPS-treated THP-1 cell-conditioned medium (CM-LPS) for 72 h. (**A**) The Cell Counting Kit-8 (CCK-8) assay was performed to measure the viability of JEG-3 and BeWo cells. The ratio of viable cells to total cells was normalized to that of the CNT group. CM-LPS treatment significantly downregulated trophoblast viability in both cell lines. (**B**) Trophoblasts were treated with culture media (CNT), CM-CNT, CM-LPS, or conditioned medium from THP-1 cells cotreated with LPS and different concentrations of ADL extract (CM-LPS+ADL) for 72 h. The CCK-8 assay was performed to measure the viability of both cell types. In both cell types, CM-LPS+ADL treatment increased trophoblast viability in a dose-dependent manner. The ratio of viable cells to total cells was normalized to that of the CNT group. (**C**) Death of JEG-3 and BeWo was analyzed by the lactate dehydrogenase (LDH) assay after treatment with culture media (CNT), CM-CNT, CM-LPS, or conditioned medium from THP-1 cells cotreated with LPS and different concentrations of ADL extract (CM-LPS+ADL) for 72 h. The ratio of LDH release was normalized to that of the lysis control. All data represent the mean ± standard deviation, n = 3. ns > 0.05, ** < 0.01, *** < 0.005, and **** < 0.001 compared with the CNT group.

**Figure 7:**
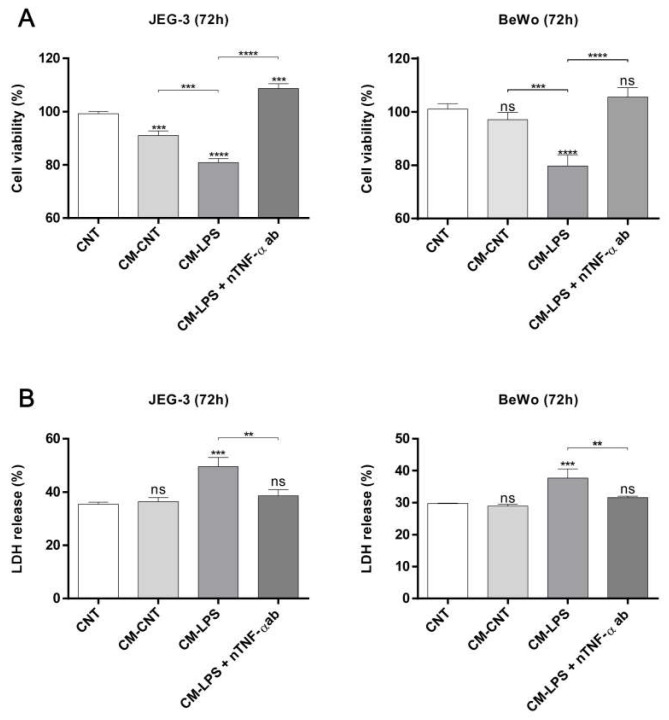
Inhibition of TNF-α rescues trophoblast death caused by LPS-treated THP-1 cell-conditioned medium. (**A**) The viability of JEG-3 and BeWo cells was analyzed by the CCK-8 assay after stimulation with THP-1-conditioned medium in the presence and absence of specific neutralizing antibodies against TNF-α. The ratio of viable cells relative to total cells was normalized to that of the CNT group. (**B**) Death of JEG-3 and BeWo was analyzed by the LDH assay after stimulation with THP-1 cell-conditioned medium in the presence and absence of specific neutralizing antibodies against TNF-α. The ratio of LDH release was normalized to that of the lysis control. Death of these cells was significantly induced by CM-LPS, but the effect is abolished after TNF-α depletion. All data represent the mean ± standard deviation, *n* = 3. ns > 0.05, ** < 0.01, *** < 0.005, and **** < 0.001 compared with the CNT group.

Proinflammatory cytokines that are secreted by activated macrophages, such as TNF-α and IL-6, have been reported to prevent trophoblast invasion and induce trophoblast apoptosis [23,24]. Additionally, we found that the TNF-α level in LPS-treated THP-1 cells is regulated by ADL extract treatment. Therefore, we next investigated whether THP-1 cell-conditioned medium influences the viability of JEG-3 and BeWo cells. Trophoblast viability decreased after exposure to LPS-treated THP-1 cell-conditioned medium, but the control-treated and untreated THP-1 cell-conditioned medium did not have any apparent effects (Figure 6A). As expected, LPS-treated THP-1 cell-conditioned medium accelerated trophoblast death in JEG-3 and BeWo cells, but this effect was abrogated after treatment with ADL extract and LPS-cotreated THP-1 cell-conditioned medium. These results indicate that trophoblast death is dose-dependently inhibited by THP-1 cell-conditioned media in the absence of LPS (Figure 6B,C). After finding that ADL extract regulates both trophoblast death induced by LPS-treated THP-1 cell-conditioned medium and LPS-induced TNF-α secretion by macrophages, we next investigated whether TNF-α is a significant factor in triggering trophoblast death. Interestingly, the death of trophoblasts decreased after TNF-α was depleted in LPS-treated THP-1 cell-conditioned medium with an anti-TNF-α antibody (Figure 7A,B). The ADL extract-induced suppression of MAPK phosphorylation and the NF-κB pathway in macrophages may indicate that ADL extract can attenuate trophoblast death by decreasing the LPS-induced secretion of TNF-α by macrophages.

### 2.6. Effect of ADL Extract on TNF-α-Induced Cell Death in Trophoblasts

**Figure 8:**
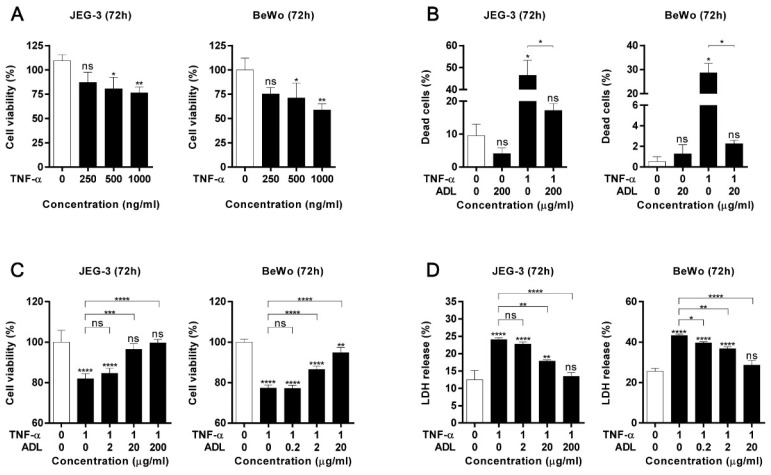
ADL extract attenuates TNF-α-induced death of trophoblasts. (**A**) The viability of JEG-3 and BeWo was analyzed by the CCK-8 assay after stimulation with different concentrations of TNF-α for 72 h. (**B**) The percentage of dead JEG-3 and BeWo cells after stimulation with TNF-α proteins and ADL extract for 72 h using the cell counter. The percentage of trypan blue-stained cells from three independent experiments is shown. (**C**) The CCK-8 assay was performed to measure the viability of both cell types. Trophoblasts were cotreated with TNF-α and different concentrations of ADL extract for 72 h. The ratio of viable cells relative to total cells was normalized to that of the CNT group. (**D**) Death of both cell types was analyzed by the LDH assay. Trophoblasts were cotreated with TNF-α and different concentrations of ADL extract for 72 h. The ratio of LDH release was normalized to that of the lysis control. All data represent the mean ± standard deviation, *n* = 3–4. ns > 0.05, * < 0.05, ** < 0.01, *** < 0.005, and **** < 0.001 compared with the untreated group.

An increase in the secretion of proinflammatory cytokines (such as the type-1 cytokines TNF-α and IFN-γ) by activated macrophages may increase the sensitivity of trophoblasts to apoptosis, which is associated with preterm birth-related inflammation [25]. Our in vivo results suggested that only TNF-α mRNA levels remained increased in the placenta at 24 h after LPS administration. Therefore, we next investigated whether ADL extract affects the TNF-α-induced death of trophoblasts. To this end, we evaluated whether TNF-α altered trophoblast viability and induced trophoblast death. Figure 8A shows that the viability of trophoblasts stimulated with TNF-α at doses of 250, 500, and 1000 μg/mL was lower than that of control trophoblasts, and that the effect of TNF-α on viability was dose-dependent. Stimulation with 1000 μg/mL TNF-α decreased the viability of both cell types. Therefore, in subsequent assays, cells were incubated with 1000 μg/mL TNF-α for 72 h. Figure 8B shows the number of trophoblasts following different treatments for 72 h. Figure 8B shows that ADL extract alone had a negligible effect on reducing the numbers of dead JEG-3 and BeWo cells (vs. CNT). The viability of JEG-3 and BeWo cells decreased after TNF-α stimulation, but this decrease was attenuated by ADL extract in a dose-dependent manner (Figure 8C). Furthermore, the LDH release levels of trophoblasts was increased after TNF-α stimulation but this increase was attenuated by ADL extract in a dose-dependent manner (Figure 8D). ADL extract was not observed to have toxic effects on either cell type at 72 h (Supplementary Figure S2C,D).

## 3. Discussion

In this study, we investigated the effect of pretreatment with ADL extract on LPS-induced preterm birth, fetal loss, and fetal growth restriction in mice. Although ADL extract alone had no effect on pregnancy outcomes, pretreatment with ADL extract markedly reduced LPS-induced fetal mortality. In addition, pretreatment with ADL extract significantly attenuated the LPS-induced reductions in fetal weight and crown–rump length. These results suggest that pretreatment with ADL extract protects against LPS-induced preterm birth, fetal loss, and fetal growth restriction. Numerous reports have shown that maternal LPS exposure during pregnancy elevates the levels of proinflammatory cytokines, including TNF-α, the major mediator of fetal death and IUGR, in maternal serum and amniotic fluid [8,26,27,28,29]. Indeed, *A. distichum* extract has been known to exhibit anti-inflammatory activity. Our results showed that pretreatment with ADL extract inhibited LPS-evoked release of the proinflammatory cytokine TNF-α in maternal serum and amniotic fluid. In addition, pretreatment with ADL extract significantly inhibited the LPS-induced upregulation of proinflammatory genes in the placenta. To our knowledge, this study demonstrates for the first time that pretreatment with ADL extract inhibits the LPS-induced inflammatory response. These results suggest that ADL extract protects against LPS-induced preterm birth, fetal loss, and fetal growth restriction partially through its anti-inflammatory activity.

According to our results, ADL extract mainly contains caffeic acid, rutin, ferulic acid, and verbascoside. These compounds have been reported to show multiple biological effects, such as antibacterial, antioxidant, anti-inflammatory, and anticancer activities [30,31,32,33]. The concentrations of rutin and verbascoside in particular are very high in ADL extract, and these two compounds have anti-inflammatory effects in various inflammatory situations [34,35,36,37,38,39,40]. Therefore, the preventive effect of ADL extract against inflammation-induced preterm birth, fetal loss, and fetal growth restriction is likely mediated by these active polyphenolic compounds.

Macrophages constitute approximately 20~30% of the immune cells at the implantation site, and the number of macrophages remains high throughout pregnancy [41,42]. The presence of an elevated number of activated macrophages that secrete proinflammatory cytokines such as TNF-α may have negative effects in pregnancy, possibly inducing conditions including miscarriage, preterm labor, preeclampsia, and IUGR [14,15,16]. Our study demonstrated that ADL extract treatment suppresses the release of TNF-α in THP-1 cells and BMDMs stimulated by LPS and that the inhibition of MAPK and IκBα phosphorylation and blockade of nuclear NF-κB accumulation underlie the effect of ADL extract.

TNF-α expression was significantly induced by LPS in THP-1-conditioned medium. JEG-3 and BeWo cell death was triggered by LPS-treated THP-1 cell-conditioned medium, and this effect was abolished after TNF-α depletion. However, various immune cells at the maternal–fetal interface, such as macrophages, natural killer cells, T cells, and dendritic cells, may produce TNF-α [43]. Other mechanisms involving different immune cells that were not assessed in our experiments may underlie TNF-α-induced trophoblast death. However, we demonstrated at least one pathway through which macrophage-secreted TNF-α affects trophoblast death. The results indicate that macrophages are involved in inflammation at the maternal–fetal interface through TNF-α expression and thus affect trophoblasts, for example, by decreasing their viability. Our data are consistent with other previous reports showing that macrophages can interact with trophoblasts to influence trophoblast phenotypes and pregnancy outcomes.

The apoptosis of villous trophoblasts occurs throughout pregnancy. Apoptosis is an essential process for placental development and may play a role in maternal immune tolerance. Higher levels of trophoblast apoptosis are observed in complicated pregnancies such as those involving pre-eclampsia or IUGR than uncomplicated pregnancies suggesting that alterations in the regulation of trophoblast apoptosis may contribute to the pathophysiology of these diseases [44,45,46,47,48,49]. TNF-α is a pleiotropic cytokine that is secreted in abundance by aberrantly activated macrophages and is capable of inducing trophoblast apoptosis. Previous studies have found that TNF-α is increased during pregnancy complications and may be involved in restricting endovascular trophoblast invasion [23,24]. Moreover, TNF-α is a cytokine that promotes inflammation and triggers cell death signaling [50]. In this study, we observed that exposure to 1 μg/mL TNF-α for 72 h induced JEG-3 and BeWo cell death. This study was designed to evaluate the effect of ADL extract in JEG-3 and BeWo cells subjected to TNF-α-induced cell death. We observed that cotreatment with ADL extract and TNF-α exerted a dose-dependent protective effect against the TNF-α-induced decrease in cell viability after 72 h. 

Collectively, the present data reveal that ADL extract may provide protective effects against LPS-induced preterm birth, fetal loss, and fetal growth restriction in mice. We found that pretreatment with ADL extract protects against the LPS-induced expression of proinflammatory cytokines, especially TNF-α, and chemokines in placental tissues through its anti-inflammatory effect. Moreover, our findings suggest that ADL extract has a potential role in attenuating the secretion of TNF-α by macrophages and TNF-α-induced trophoblast death. In conclusion, our data provide new evidence identifying ADL extract as a therapeutic candidate for inflammation-induced preterm birth, fetal loss, and fetal growth restriction due to its anti-inflammatory effect.

## 4. Materials and Methods

### 4.1. Plant Materials and HLPC Analysis

*A. distichum* leaf (ADL) ethanolic extract was obtained from the Korea Prime Pharmacy Co., Ltd. located in Gwang-ju metropolitan city, South Korea. Dried ADL was obtained from Woorinamoo Agricultural Union Corporation (Goesan, Republic of Korea). Two kilograms of dried and crushed ADL were extracted for 4 h at 75 °C using 40 L of 50% ethanol, then filtered through filter paper (No. 4, Whatman international Ltd., Springfield Mill, Kent, UK). The filtrate containing solvent and water was evaporated to two liters by using a rotary evaporator (EYELA N-3010; Tokyo, Japan) at 50 ℃. The residue was lyophilized (Freeze dryer, FD8508, IlShinbBioBase Co. Ltd, Gyeonggi, Korea) and the amount of extract of ADL extract was then 1.02 kg (extraction yield 50%). The lyophilized powder was stored at −20 °C until its use.

The HPLC system used was a Shimadzu liquid chromatography system (LC-20AD) using a diode array detector (SPD-M20A) and YMC Triart C18 column (4.6 × 250 mm, 5 μm). The mobile phase consisted of solvents A and B. Solvent A was 0.1% trifluoroacetic acid in water, and solvent B was 0.1% trifluoroacetic acid in acetonitrile. The gradient was 0 min, 10% B; 0–20 min, 20% B; 20–30 min, 20% B; 30–35 min, 100% B; 35–40 min, 100% B; 40–41 min, 10% B; 41–50 min, 10% B. The run time was 50 min using a flow rate of 1.0 mL/min. The phenolic compounds were identified by the retention time and UV spectrum of the standard measured from the peak area at 254 nm. The concentration was calculated by comparing the peak areas of the samples with the calibration curve of the standards.

### 4.2. Mice

C57BL/6J (8 weeks) mice were purchased from DBL (Chungcheongbuk-do, Korea). The animals were kept in an specific pathogen free (SPF) barrier room under controlled conditions of a 12-h light–dark cycle and a constant temperature (25 °C). Animal Care and the Guiding Principles for Animal Experiment Using Animals were approved by the University of Konyang Animal Care and Use Committee (20-02-E-02). For mating purposes, one male mouse (8~10 weeks old) and one female mouse (8 weeks old) were individually housed together starting at 9:00 PM. The next morning, the vaginal plug of the female mouse was checked and was considered as GD 0. Pregnant mice were randomized into four groups.

### 4.3. Animal Experiments

In this experiment, lipopolysaccharides (LPSs) were treated to discover the efficacy of ADL ethanolic extract in pregnancy outcomes according to inflammatory environments. The LPS from the *Escherichia coli* O111:B4 (Sigma-Aldrich, St. Louis, MO, USA) used were dissolved in phosphate-buffered saline (PBS). The dose of the LPS used in this study referred to other studies [51,52]. Pregnant mice were i.p. injected with LPS (40 μg/kg) on GD 16 to evaluate the occurrence of preterm birth, fetal loss, fetal growth restriction, and intrauterine fetal death. Mice were monitored for preterm birth rates after injection (10 to 12 mice). At GD 19, pregnant mice were sacrificed and then the number of live fetus and dead fetus sites were counted (4 to 5 mice). Live fetuses and placentas were weighed and the crown–rump length was measured (4 to 5 mice). To investigate the effects of ADL on LPS-induced placental inflammation, pregnant mice were i.p. injected with LPS (40 μg/kg) on GD 16 (3 to 4 mice). All dams were sacrificed either 1 or 24 h after LPS injection. Amniotic fluid and serum were collected from all mice and stored at –80 °C. Placentas were collected for real-time RT-PCR and stored in the N₂ tank. ADL was orally administered daily for 15 days at a dose of 100 μg/kg/day dissolved in autoclaved water.

### 4.4. Cell Culture

Human trophoblastic cells JEG-3 and BeWo cells were purchased from the American Type Culture Collection (ATCC; Manassas, VA, USA). Monocytic THP-1 cells were purchased from the Korean Cell Line Bank (Seoul, Korea). All cells were grown in Dulbecco Modified Eagle Medium (DMEM; Biowest, Riverside MO, USA) and were all supplemented with 10% fetal bovine serum (FBS; Biowest), Antibiotic/antimycotic Solution (Welgene, Daegu, Korea). The cells were incubated at 37°C and 5% CO₂.

THP-1 cells were differentiated into macrophage-like cells by incubation with 100 nM phorbol myristic acetate (PMA; Sigma-Aldrich) overnight. The stimulators used in this study were LPS (Sigma-Aldrich), recombinant human tumor necrosis factor-α (JW CreaGene, Gyeonggi-do, Korea), and TNF-α monoclonal antibody (Invitrogen, Carlsbad, CA, USA). Before their use for treatment, all compounds were dissolved in Dulbecco’s Phosphate-Buffered Saline (DPBS; Biowest).

### 4.5. Generation of Mouse Bone Marrow-Derived Macrophages

Bone marrow-derived macrophages (BMDM) were isolated from the marrow of the femurs and tibias of 6 weeks old C57BL/6 mice. The legs of the animals were sprayed with 70% EtOH, and the skin and muscle tissue were removed from the bones. The bones were sprayed with 70% EtOH, transferred to a sterile-flow hood, and cut at both ends. The marrow was flushed out into a sterile falcon tube in fresh DMEM. The cell suspension was triturated using a 1 mL syringe, filtered through a 40 μm Sterile Cell Strainer (BD falcon, Franklin Lake, NJ, USA) into a 50 mL sterile tube and centrifuged (1500 rpm, 3 min). The supernatant was discarded and the cells were washed using DMEM and centrifuged once more (400× *g*, 5 min). The pellet was resuspended in 40 mL of DMEM supplemented with 10 ng/mL recombinant mouse M-CSF (JW CreaGene). Cells were seeded in 100 mm TC-treated Culture Dish (Corning, Corning, NY, USA). On day 3, the media were replaced and maintained in culture for a further 3 days.

### 4.6. Conditioned Medium Preparation

THP-1 (1 × 10⁶ cells/well) was grown in 6-well plates. The cells were washed with phosphate-buffered saline (PBS) and incubated in regular medium containing FBS with or without LPS and ADL stimulation. At the end of treatment for 24 h, the conditioned medium of LPS-stimulated cells was harvested in a 50 mL conical tube and centrifuged at 3000 rpm to eliminate intact cells, and the supernatants were stored at −80 °C until use.

### 4.7. Cell Viability and Cytotoxicity Assay

The proportion of viable cells was determined using a Cell Counting Kit-8 (Dojindo, Kumamoto, Japan) according to the manufacturer’s protocol. Treated cells in 96-well plates were washed with PBS and changed to a fresh medium with 10% CCK-8. Then, the plates were incubated for an additional 1.5 h at 37 °C. The absorbance of staining intensity in the medium was measured in 450 nm wavelengths using the microplate spectrophotometer (Epoch, BioTek, USA).

The level of cytotoxicity was assayed using the LDH Cytotoxicity Assay Kit (DoGenBio, Seoul, Korea), which evaluates cytotoxicity by measuring the amount of lactate dehydrogenase (LDH) released from the cytosol of damaged cells according to the manufacturer’s instructions. To quantitate the LDH level, the supernatant of culture samples (10 μL) and the LDH Reaction Mixture (100 μL) were mixed in a 96-well plate, and then incubated in dark at room temperature for 30 min. In order to measure the maximum LDH level, the lysis solution was added to the control cells and incubated for 5 min at 37 °C, and then the supernatant was used after centrifuged at 600× *g* for 5 min. The absorbance of staining intensity in the medium was measured in 450 nm wavelengths using the microplate spectrophotometer (BioTek) in three independent experiments. The percentage of LDH released (%) was calculated using the formula (percentage cytotoxicity) = (OD450 of experimental group)/(OD450 of high control) × 100.

### 4.8. Quantification of mRNA by Real-Time RT-PCR

Total RNA was isolated from the placenta tissues of mice using an AccuPrep™ Universal RNA Extraction Kit (BIONEER, Daejeon, Korea). Total RNA was prepared according to the manufacturer’s protocol. Briefly, 500 ng of total RNA was used as the template for single-strand cDNA synthesis with a PrimeScript RT Master Mix (Takara, Tokyo, Japan). Real-time PCR was performed using the LightCycler® 480 SYBR® Green Ⅰ Master (Roche, Basel, Switzerland) on a CFX Connect™ Real-Time System, and results analyzed with the CFX Maestro™ Software 1.1. The real-time PCR started with an initial enzyme activation step (10 min, 94 °C), followed by 40 or 50 cycles consisting of a denaturing (30 s, 94 °C) and an annealing/extending (30 s, 60 °C) step. The primer sets used were as follows: mouse IL-1β (forward: GCCCATCCTCTGTGACTCAT; reverse: AGGCCACAGGTATTTTGTCG), IL-6 (forward: GATGGATGCTACCAAACTGGAT; reverse: CCAGGTAGCTATGGTACTCCAGA), IL-12p40 (forward: GGAAGCACGGCAGCAGAATA; reverse: AACTTGAGGGAGAAGTAGGAATGG), IL-10 (forward: GGTTGCCAAGCCTTATCGGA; reverse: ACCTGCTCCACTGCCTTGCT), TNF-α (forward: TCTTCTCATTCCTGCTTGTGG; reverse: GGTCTGGGCCATAGAACTGA), CCL-2 (forward: GCTCAGCCAGATGCAGTTAAC; reverse: CTCTCTCTTGAGCTTGGTGAC), CCL-3 (forward: TGCCCTTGCTGTTCTTCTCT; reverse: GTGGAATCTTCCGGCTGTAG), CCL-4 (forward: CATGAAGCTCTGCGTGTCTG; reverse: GGAGGGTCAGAGCCCATT), CXCL-1 (forward: ATCCAGAGCTTGAAGGTGTTG; reverse: GTCTGTCTTCTTTCTCCGTTACTT) and F4/80 (forward: CTTTGGCTATGGGCTTCCAGTC; reverse: GCAAGGAGGACAGAGTTTATCGTG). The amount of RNA was normalized to the β-actin signal amplified in a separate reaction (forward: TACCCAGGCATTGCTGACAGG; reverse: ACTTGC- GGTGCACGATGGA).

### 4.9. ELISA

Mouse serum and amniotic fluid samples were collected and centrifuged at 3000 rpm for 5 min. Conditioned medium from THP-1 and BMDM were collected after a 24-h incubation and centrifuged at 3000 rpm for 5 min. Samples were stored at −80 °C until analysis. For TNF-α detection, the human or mouse cells conditioned medium and the mouse serum and amniotic fluid were assessed by ELISA kits (Invitrogen) according to the manufacturer’s protocol. Microtiter plates were incubated overnight at 4 °C with 100 µL/well of capture antibody in coating buffer. The wells were then washed 3 times with PBS containing 0.05% Tween-20 (LPS solution, Daejeon, Korea) and were blocked with 200 µL of ELISA/ELISPOT Diluent (1X) for 1 h. Subsequently, each well was loaded with 100 L of samples and standards and was incubated for 2 h at room temperature. The wells were then incubated with detection antibody and streptavidin-HRP in ELISA/ELISPOT diluent (1×) for 30 min at room temperature in the dark. Finally, the plates were treated with 3,3’,5,5’-tetramethylbenzidine (TMB) solution (1×) for 30 min, and the reaction was stopped by the addition of stop solution. The absorbance of optical density was measured in 450 nm wavelengths using the microplate spectrophotometer (BioTek). The values were then calculated from the standard curve.

### 4.10. Western Blot

THP-1 and BMDM were lysed in RIPA buffer (iNtRON, Gyeonggi-do, Korea) containing Protease Inhibitor Cocktail (Roche), and lysates were centrifuged (12,000 rpm, 20 min, 4 °C). The supernatant was collected and stored at −80 °C. The protein concentrations of the lysates were determined using the Protein Assay Dye Reagent Concentrate (Bio-Rad, Berkeley, CA, USA). The protein was mixed with 5X SDS-PAGE Loading Buffer (LPS solution) and denatured by heating to 100 °C for 5 min. Then, the samples were electrophoresed in 12% Tris-glycine gels and transferred to 0.45 μm nitrocellulose membranes (Bio-Rad). The membranes were blocked with 5% skim milk (Difco, Detroit, MI, USA) prepared in TBS (LPS solution) or PBS (LPS solution) containing 0.1% TWEEN 20 and then incubated overnight at 4 °C with the following antibodies: rabbit anti-IκB-α; rabbit antiphospho-IκB-α; rabbit anti-phospho-p38 MAPK; rabbit anti-p38 MAPK; rabbit anti-phospho-ERK1/2; rabbit anti- ERK1/2; rabbit anti-phospho-JNK; rabbit anti-JNK (1:1000; CST, Boston, MA, USA); mouse anti-β-actin (1:2500; Sigma-Aldrich). Next, the immunoblots were washed in TBST, incubated with a horseradish peroxidase-conjugated secondary antibody (1:20,000; CST) for 1 h at room temperature, and treated with EZ-Western Lumi Femto (DoGenBio). The images were observed with a Fusion Solo S (VILBER, France) and analyzed with Vision-Capt software (VILBER).

### 4.11. Immunofluorescence

BMDMs were cultured on Poly-D-Lysine-coated (GIBCO, Grand Island, NY, USA) cover glasses in 24-well plates at a density of 2 × 10⁵ cells/well, fixed in 3.7% formaldehyde solution (Sigma-Aldrich) for 15 min, washed with PBS and permeabilized with 0.05% Triton-X-100 (Sigma-Aldrich) for 10 min, then blocked with CAS-Block Histochemical Reagent (Invitrogen) for 30 min, all at room temperature. Subsequently, the cells were incubated with p65 primary antibody (1:500; abcam, Cambridge, UK) overnight at 4 °C, followed by incubation with an Alexa Fluor^®^ 488 conjugated anti-Rabbit IgG Secondary Antibody (Invitrogen) in dark at room temperature for 1 h. Cells were then incubated in the dark with DAPI (1:1000; Sigma-Aldrich) and Texas Red™-X Phalloidin (Invitrogen) for 10 min. After washing with PBS, cover glasses were mounted with VECTASHIELD Vibrance Antifade Mounting Media (VECTOR, Burlingame, CA, USA). Images were recorded using a Leica confocal microscope (Leica Microsystems, Germany) and the Leica Application Suite Advanced Fluorescence software.

### 4.12. Assessment of Dead Cell Count

To assess dead cell count, trophoblast cells exposed to TNF-α and ADL were reseeded in triplicates into 6-well plates. After 72 h, cells were harvested with Trypsin 0.25% EDTA in HBSS (Biowest) and enzymatic activity was neutralized with FBS-containing DMEM media. The cells were then washed with PBS and were stained with 0.4% Trypan Blue Solution (GIBCO). The trophoblast cell count was then quantified by counting the viable and dead cells using the CytoSMART cell counter (Corning). 

### 4.13. Statistical Analysis

All figures analyses with three or more treatment groups were analyzed with a one-way ANOVA, followed by Tukey’s multiple comparisons test or Chi-square test. The data are presented as means ± SD. GraphPad Prism® version 6.01 (GraphPad Software, San Diego, CA, USA) was used for all calculations. Statistical significance was denoted with P value of less than 0.05.

## Figures and Tables

**Table 1:** The five major phenolic compounds in *Abeliophyllum distichum* leaf (ADL) ethanolic extract.

Phenolic Compounds	Contents (μg/mg)
Chlorogenic acid	11.60 ± 0.02
Caffeic acid	11.26 ± 0.02
Rutin	32.72 ± 0.01
Ferulic acid	20.70 ± 0.04
Verbascoside	260.43 ± 0.10
Mean ± SD (*n* = 3)	

## Supplementary Materials

The following are available online, Figure S1: ADL extract pretreatment prevents LPS-induced expression of proinflammatory cytokines and chemokines, Figure S2: Effects of ADL extract on macrophage and trophoblast viability.
