# Supplementary material for: Verbascoside-Rich Abeliophyllum distichum Nakai Leaf Extracts Prevent LPS-Induced Preterm Birth Through Inhibiting the Expression of Proinflammatory Cytokines from Macrophages and the Cell Death of Trophoblasts Induced by TNF-α
Source: Molecules. 2020 Oct 7;25(19):4579. doi: 10.3390/molecules25194579 (PMC7583932; doi:10.3390/molecules25194579)
Supplement: Supplementary file 1 [file molecules-25-04579-s001.zip › Supplemental Figure Legends.docx]

**Supplemental Figure Legends**

**Supplemental Figure 1. ADL extract pretreatment prevents LPS-induced expression of proinflammatory cytokines and chemokines.** In the ADL alone and LPS+ADL groups, pregnant mice were gavaged with ADL extract (100 μg/kg) daily from GD1 to GD16. In the LPS alone and LPS+ADL groups, pregnant mice were i.p. injected with LPS (40 μg/kg) on GD 16. Mouse placental tissues were collected 24 h after LPS (40 μg/kg) injection. Placental samples were collected from maternal mice from each group, pooled, and used for analysis. Relative mRNA expression levels of (A) TNF-α, (B) IL-1β, (C) IL-6, (D) IL-12p40, (E) CXCL-1 and (F) F4/80 in the placenta were measured by real-time RT-PCR and normalized to the level of β-actin. Maternal serum was collected 24 h after LPS (40 μg/kg) injection. (F) The concentration of TNF-α in maternal serum was measured using ELISA. All data represent the mean ± standard deviation, n=3-4. ns > 0.05, * < 0.05, ** < 0.01, *** < 0.005, and **** < 0.001 compared with the CNT group.

**Supplemental Figure 2. Effects of ADL extract on macrophage and trophoblast viability.** (A, B) Cell viability was measured by the CCK-8 assay after treatment with ADL extract. The viability of THP-1 cells and BMDMs was measured by the CCK-8 assay after 24 h of incubation with different concentrations of ADL extract. (C, D) The viability of JEG-3 and BeWo cells was measured by the CCK-8 assay after 72 h incubation with different concentrations of ADL extract. All data represent the mean ± standard deviation, n=3. * < 0.05, ** < 0.01, and *** < 0.005 compared with the untreated group.

Table 1. The five major phenolic compounds in ADL ethanolic extract.
