# Supplementary figures and images for: Verbascoside-Rich Abeliophyllum distichum Nakai Leaf Extracts Prevent LPS-Induced Preterm Birth Through Inhibiting the Expression of Proinflammatory Cytokines from Macrophages and the Cell Death of Trophoblasts Induced by TNF-α
Source: Molecules. 2020 Oct 7;25(19):4579. doi: 10.3390/molecules25194579 (PMC7583932; doi:10.3390/molecules25194579)

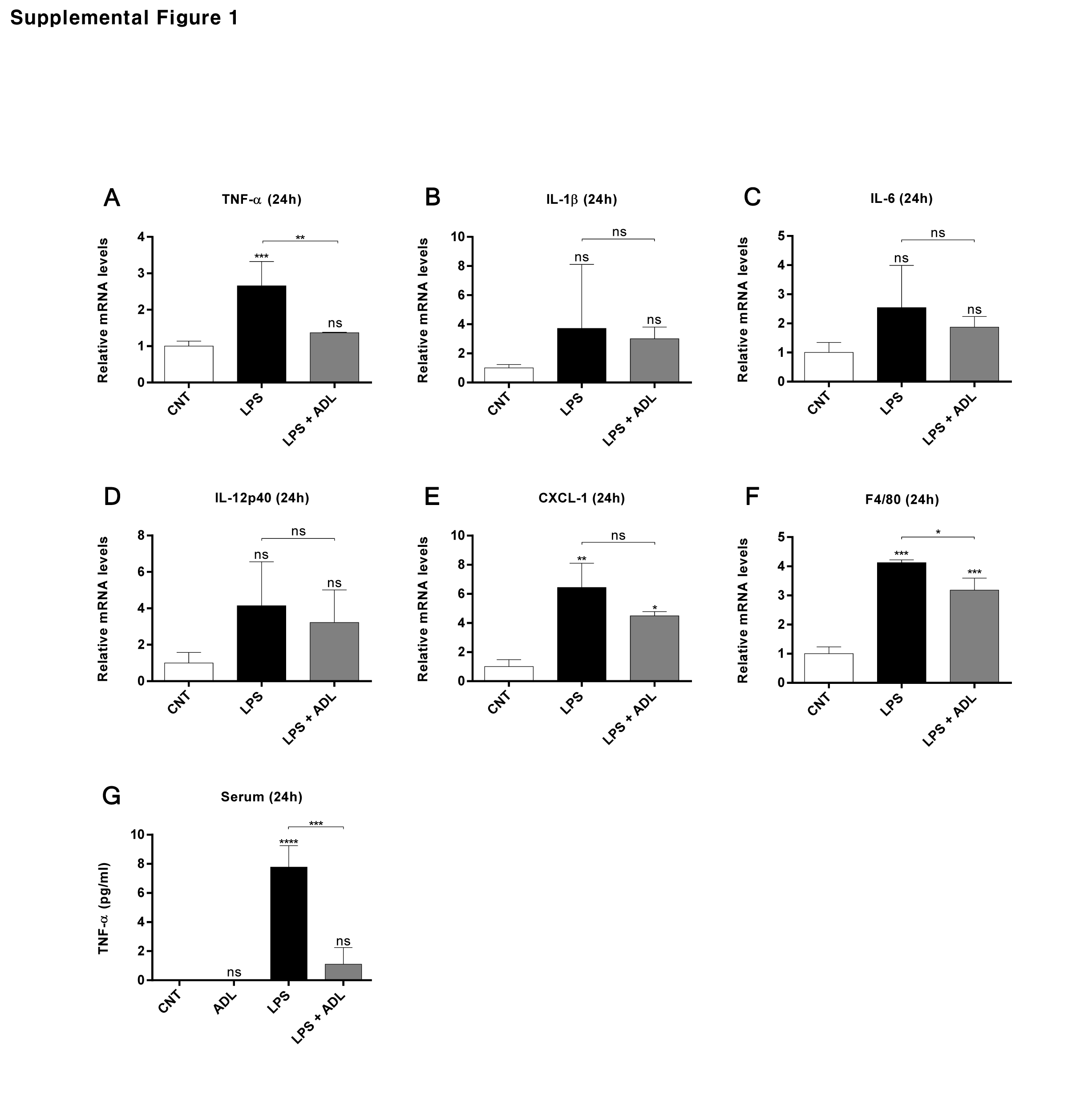

Supplement: Supplementary file 1 [file molecules-25-04579-s001.zip › S1.tif]

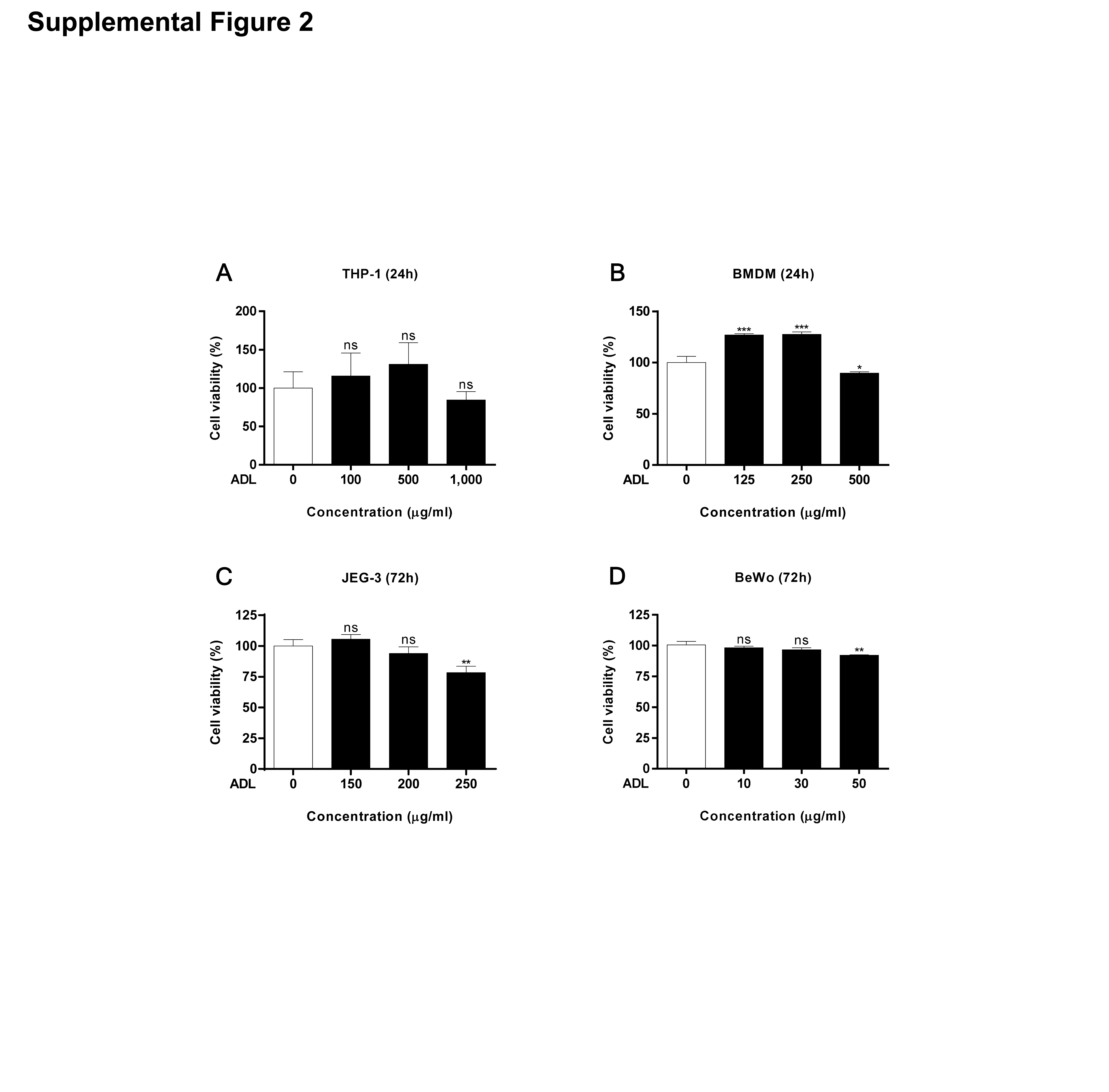

Supplement: Supplementary file 1 [file molecules-25-04579-s001.zip › S2.tif]
